# Supplementary material for: Characterization of endoplasmic reticulum-associated degradation in the human fungal pathogen Candida albicans
Source: PeerJ. 2023 Aug 25;11:e15897. doi: 10.7717/peerj.15897 (PMC10461541; doi:10.7717/peerj.15897)
Supplement: Supplemental Information 1 [file peerj-11-15897-s001.docx]

**Supplemental File 1.** CLUSTAL multiple sequence alignment of amino acid sequence of Hrd1 homologs in *H. sapiens*, *S. cerevisiae*, and *C. albicans*.

HsHRD1 --MFRTAVMMAASLALTGAVVAHAYY-LKHQFYPTVVYLTKSSPSMAVLYIQAFVLV--F 55

ScHRD1 MVPENRRKQLAI**FVVVTYLLTFYCVYSATKT**SVSFLQVTLKLNEGFNLM**VLSIFILLNST** 60

CaHRD1 MLINKTQQYIIGYSSISIALLSWSIYDSLRKSFNFVMFLVEFTDGIKLGIIVNFIIFLFL 60

.. .. . .. .: * . .. .: .. . . .. : : *::.

HsHRD1 LLGKVMGKVFFGQLRAAEMEHLLERSWYAVTETCLAFTVFRDDFSPRFVALFTLLLFLKC 115

ScHRD1 **LLWQLLTKLLF**GELRLIEHEH**IFERLPFTIINTLFMSSLFHER**YFFT**VAFFGLLLLYLKV** 120

CaHRD1 IIDKLLQILLFGSLRMIEVEHLFEKLPIFVINLLLNLATGDNNIIMNVFLMGLSMSF-KV 119

:: ::: .::** ** .* **::*:. . :....: . . . . . ..:.: *.

HsHRD1 FHWLAEDRVDFME----RSPNISWLFHCRIV-----SLMFLLGIL----DFLFVSHAYHS 162

ScHRD1 **FHWILKDRLEALL----Q**SINDSTTMKT**LIFSRFSFNLVLLAVV-----DYQIITRCISS** 171

CaHRD1 FHVIMFDRLDYVNLIIVNKINDEDIYLNQVIYHFGTSINFWLNIFFIFADFSLAKFLVYD 179

**.: **:: . ...*.. .. :. .: :.. : *: .. ..

HsHRD1 ILTRGAS------VQLVFGFEYAILMTMVLTIFIKYVL---------------------- 194

ScHRD1 **IYTN**QKSDIE**STSLYLIQVMEFTMLLIDLLNLFLQTCL-------------------NFW** 212

CaHRD1 VFQGINS-----VTCLLFGFQFAVQGVQALTYFSKLLLGIYEIAFYRIRKNEHHTLLRGW 234

:.. * *....:::.. . .*. * : *

HsHRD1 ------------------------HSVDLQSENP-------------------------- 204

ScHRD1 EFYRSQQSLSNENNHIVHGDPTDENTVESDQSQPVLNDDDDDDDDDRQFTG--------- 263

CaHRD1 QESTTTSANLEETERTTNNNDNESGQVEVEVEANIDEDVDVDADADADVDVEEEIVNDED 294

*: . . .

HsHRD1 ------WDNKAVYMLYTELFTGFIKVLLYMAFMTIMIKVH---TFPLFAIRPMYLAMRQF 255

ScHRD1 ------**LEGKFMYEKAIDVFTRFLKTALHL**S---MLIPFR----MPMMLL**KDVVWDILAL** 310

CaHRD1 DDIELIWDNKPYYTKGIDIASAVLTSISYLSFV-YLLTIHSGLSLPLSMLQGTYSSMRRA 353

.:.* * . .:... .:. .::. :: .: .*: :. .. :.

HsHRD1 KKAVTDAIMSRRAIRNMNTLYPD**ATPEELQAM---DNVCIICREEMVT------------** 300

ScHRD1 **YQSGTSLWKIWR**NNKQLDDTLVT**VTVEQLQNSANDDNICIICMDELIHSPNQQTWKNKNK** 370

CaHRD1 WVETNQLLAFIESSKRLDTQLAN**ASAEDLSQS---DSLCIICREDMHSVEDYQRIFKKPQ** 410

. . .. :.::. . ... *.*. . *..****.:::

HsHRD1 **GAKRLP----CNHIFHTSCLRSWFQRQQTCPTCRMDVL**RASLPAQSPPPPEPADQGPPPA 356

ScHRD1 **KPKRLP----CGHILHLSCLKNWMERSQTCPICRLPVF**-----------DEKGNVVQTTF 415

CaHRD1 **SPRRSPKKLKCGHILHLGCLKEWLERSDSCPLCRRKVF**--------------SNDGATNA 456

.:*.* *.**:*..**: *.:*...**.** *: .: . . .

HsHRD1 PHPPPLLPQPPNFPQGLLPPFPPGMFPLWPPMGPFPPVPPPPSSGEAVAPPSTSAALSRP 416

ScHRD1 TSNSDITTQTTVTDSTGIATDQQGFANE---------VDLLPTRTTSPDIRIV------P 460

CaHRD1 TNNNNNNNGENNQNNGNVPPPQPGDVPV---------PPPAPTATA**TATAAAVNAELQRE** 507

. . . . . ... ..* . ... *. . . . .

HsHRD1 SGAATTTAAGTSATAASATASGPGSGSAPEAGPAPGFPFPPPWMGMPLP------PPFAF 470

ScHRD1 TQNIDTLAMRTRSTSTPS----------------------PTWYTFPLHKTGDNSVGSSR 498

CaHRD1 **VREINDLLQNAENT**IAPQEQP-INTAAEPIEGPSRTIPLPSSSSTLPIPQSNDLSQSITL 566

..... : .* :. ... ..*:. .

HsHRD1 PPMPVPPAGFAGLTPEELRALEGHER**QHLEARLQSLRNIHTLLDAAMLQINQYLTVLAS**L 530

ScHRD1 SAYEFL--------------ITNSDEKENGIPVK-------------LTIEN-HEVNSLH 530

CaHRD1 PKNALLPPGWLVI------PLKKPEQENSDIDYK-------------VNISSYHQADLKI 607

. .. : :... .. : : * . .

HsHRD1 GPPRPATSVNSTEETATTVVAAASSTSIPSSEATTPTPGASPPAPEMERPPAPESVGTEE 590

ScHRD1 GD--------GGEQIAKKIV-------------------------------IPDKFIQHI 551

CaHRD1 NNKKP-----STRDIITYAI------------------------------PKEESIGEYE 632

. ....... .: .:... .

HsHRD1 MPEDGEPDAAELRRRRLQKLESPVAH 616

ScHRD1 -------------------------- 552

CaHRD1 M------------------------- 633

**KEY**

Gray Highlight = Transmembrane portion of protein

**Black bold** = approximate positions of 8 Sc Hrd1 transmembrane segments (per Wu et al. 2020)

**Red bold** = catalytic RING domain

**Green bold** = Metal (zinc)-coordinating Cys and His residues in RING domain

Yellow highlight = Trp commonly found in RING domains

Turquoise highlight = C-terminal extension (downstream of RING); important for forming ERAD complex formation (Schulz 2017) and binding at least a subset of substrates (Omura et al. 2006)

**Orange bold** = predicted α helix within C-terminal extension – present in Ca and Hs, but not Sc; important for ERAD complex formation in human complex (Schulz et al. 2017)

Produced using SnapGene.
