# Supplementary material for: Characterization of endoplasmic reticulum-associated degradation in the human fungal pathogen Candida albicans
Source: PeerJ. 2023 Aug 25;11:e15897. doi: 10.7717/peerj.15897 (PMC10461541; doi:10.7717/peerj.15897)
Supplement: Supplemental Information 2 [file peerj-11-15897-s002.docx]

**Supplemental File 2.** CLUSTAL multiple sequence alignment of amino acid sequence of Doa10 homologs in *H. sapiens*, *S. cerevisiae*, and *C. albicans*.

HsTEB4 ------------------------------MDTAEEDI**CRVCRSEGTPEKPLYHPCVCTG** 30

ScDoa10 MDVDSDVNVSRLRDELHKVANEETDTATFNDDAPSGAT**CRICRGEATEDNPLFHPCKCRG** 60

CaDoa10 -------------------------------MSSTDHT**CRICRGEATSSQPLYHPCKCRG** 29

... . .**:**.*.* ..**:***.*.*

HsTEB4 **SIKFIHQECLVQWLKHSRKEY--------CELCKHRFA**FTPIYSPDMPSRLPIQDIFAGL 82

ScDoa10 **SIKYMHESCLLEWVASKNIDISKPGADVKCDICHYPIQ**FKTIYAENMPEKIP**FSLLLSKS** 120

CaDoa10 **SIKYIHQDCLMEWLKHSNKSTEK------CDICNSPYK**FKIIYDPAMPQYIPLDLIWKKF 83

***::*:.**.:*:...... *::*.... *. ** . ** :*. : .

HsTEB4 VTSIGTAIRYWFHYTLVAFAWLGVVPLT---ACRIYKCLFTGS--------VSSLL---- 127

ScDoa10 **ILTFFEKARLALTIGLAAVLYIIGVPLVWNMFGKLYTMMLD**GSSPYPGD-FLKSLIYG-- 177

CaDoa10 LQITSSTVFKSISISLYILCIVIQVPLFWKFSGRVYTWAIDGTLPLVNQKFVDALLFGEF 143

. .. . ..* .. .. *** .:.*. ..*. : .*:

HsTEB4 ---TLPLDMLSTENLLA----------DCLQGCFVVTCTLCAFISLVWLREQIVHGGAPI 174

ScDoa10 ------**YDQSATPELTTRAIFYQLLQNHSFTSLQFIMIVIL-HIALYFQYDMIVR**EDVFS 230

CaDoa10 DINTYLADKLQTPLQLSLMKLKKFFGCTYFSGVRYLFVAIVANIALFIEREWVVRDEGYL 203

* . *. ... .: . . .: *.* .: :*:..

HsTEB4 ------------------------------WLEHAAPPFNAAGHHQNEAPA--------- 195

ScDoa10 KMVFHKIGPRLSPKDLKSRLKERFPMMDDRMVEYLAREMRAHDENRQEQGH---DRLNMP 287

CaDoa10 KMLHKKIG-----KEHRAKLVD--------MLQNALQGLRTDGNNGDEAAAANLQRLETL 250

.::... ..: ... .*. .

HsTEB4 --GGNGAENV-------------------------------------AADQPANPPAENA 216

ScDoa10 AAAADNNNNV-----INPRNDNVPPQDPNDHRNFENLRHVDELDHDEATEEHENNDSDNS 342

CaDoa10 ATAINDLQNEDRDMNVAARGEAALRRAIDQNQLFGNEQQPILNQFENGIGQHDALPTHNF 310

. :. .*. ...:. . ...*

HsTEB4 VVGENP-----------DAQDDQAEEEEEDNEEEDDAGVEDAAD---------------- 249

ScDoa10 LPSGDDSSRILPGSSSDNEEDEEAEGQQQQQQPEEEADYRDHIEPNPIDMWANRRAQNEF 402

CaDoa10 DAPENLMNGVFNGRNDIGSDDEEDEEQQEEEDDDEEAAVEEDVD---------------- 354

.: . .*::.*.:::... :::* ..: .:

HsTEB4 ------------------------ANNGAQDDMNWNALE------------WDRAAEELT 273

ScDoa10 DDLIAAQQNAINRPNAPVFIPPPAQNRAGNVDQDEQDFGAAVGVPPAQANPDDQGQGPLV 462

CaDoa10 -----------------VAAAAAAGADGGVVGEFLEAFG--------------------- 376

. .. .. ..:.

HsTEB4 WERMLGLDGSLVFLEHVFWVVSLNTLFILVFAFCPYHIGH---------FSLV--GLGFE 322

ScDoa10 I**NLKLKLLNVIAYF---IIAVVFTAIYLAISYLFPTFIGFGLLKIYFGIFKVILRGLCHL** 519

CaDoa10 --VTLTLSTPIYLM---FLCNCIVAVYLFLIYLIPHMLGNTIVSITTFLFKLI--NVTLI 429

* * : .. . . . :.:: . .: * :* *.:: .:

HsTEB4 EHVQASH---------------------------------FEGLITTIVGYILLAITL-- 347

ScDoa10 **YYL**SGAHIAYNGLTKLVPKVDVAMSWISDHLIHDIIYL--YNGYTENTMKHS**IFIRALPA** 577

CaDoa10 SYIS-KHIPFP-------------TYIYEFASLYITSLGTFNPNKGVTLIERIFILGL-- 473

:.. * :.. .. ::. .*

HsTEB4 ---------IICHGLATLVKFHRS---------RRLLGVCYIV---VKVSLLVVVEIGVF 386

ScDoa10 **LTTYLTSVSIVCASSNLVSR**GYGRENGMSN**PTRRLIFQILFALKCTFKVFTLFFIELAGF** 637

CaDoa10 ------GYGLICTTIYRLMKFLVSGPKPISGTPRKAFKVLFEISSTAKVFLIFAIEIFFF 527

::* . : : . * : :.: . **..:. :*: *

HsTEB4 PLICGWWLDICSLEMFDATLKDRE--------------LSFQSAPGTTMFLHWLVGMVYV 432

ScDoa10 **PILAGVMLDFS---LFCPIL**ASNSR-------MLWVPSICAIW**PPF-SLFVYWTIGTLYM** 686

CaDoa10 PVYCGWLLDFCAAPLFVPQFIQTAENGGKVVTFLVSSYFEMMQLPYLRVLLYWASGTLYM 587

*. .*. **.. :* . : . * .:::* .*.:*:

HsTEB4 FYFASFILLLRE-VLRPGVLWFLRNLNDPDFNPVQEMIHLPIYRHLRRFILSVIVFGSIV 491

ScDoa10 **YWFAKYIGMIRKNI**IRPGVLFFIRSPEDPNIKILHDSLIHP**MSIQLSRLCLSMFIYAIFI** 746

CaDoa10 LFFALYIGMVRSTILRPGVLFFIRCPDDPNTRLIHDALVKPLSLQLSRIYLTAKVYSAFI 647

..** :* :.* ::*****.*:* ..**: . .:: : *.. :*.*. *.. ::. .:

HsTEB4 LL------MLWLPIRIIKSVLPNFLPYNVMLYSDAPVSELSLELLLLQVVLPALLE-QGH 544

ScDoa10 **VLGFGFHTRIFFPFM**LKSNLLSVPEAYKP-TSIIS**WK---FNTILLTLYFTKRILES**SSY 802

CaDoa10 IFGIGG--VTW---GLRYLVTPKDKDYNVFLPIQTPS---YFTYLLLAIIVPTLVDSQAT 699

.: .. : :.. *.. . . . . **. . . ::: ..

HsTEB4 TRQWLKGLVRAWTVTAGYLLDLHSYLLGDQEENENS------------------ANQQVN 586

ScDoa10 VKPLLERYWKTIFKLCSRKLRLSSFILGKDTPTERGHIVYRNLFYKYIAAKNAEWSNQEL 862

CaDoa10 VTKYVRQYWERAFEISSHKLRLSHFIVGKPISSERGYVIYRNIWLQLFG-----LNVQPD 754

.. :. . . . ...*.*..:::*. .*.. . *

HsTEB4 NNQHARNNNAIPVVGEG------------------------------------------- 603

ScDoa10 FTKPKTLEQAEELFGQVRDVHAYFVPDGVLMRVPSSDIVSRNYVQTMFVPVTKDDKLLKP 922

CaDoa10 YTQPVTRKEALAKFKQDPNAVAFFVPDGNYVRAPANDTISRKFVKKLFVAVSKDDRL--- 811

.:. . ..* ..:

HsTEB4 --LHAAHQAILQQGGPVGFQPYR----------------RPLNFPLRIFLLIVFMCITLL 645

ScDoa10 LDLERIKERNKRAAGEFGYLDEQN-----TEYDQYYIVYVPPD**FRLRYMTLLGLVWLFAS** 977

CaDoa10 --LQEVKEAPKRSGYETPTSDEDEDTTTTTTDDAYTIVYRPPNFKLRCFALILMLWIFAI 869

*. ..:. .: ... . . .*.:* ** . *: ...:..

HsTEB4 IASLICLTLPVFAGRWLMSFWTGTAKIHELYTAACG-LYVCWLTIRAVT---VMVAWMPQ 701

ScDoa10 **ILMLGVTFISQALINFVCSF--GFLPVVKLLL**GERNKVYVAWKELSDISYSYLN**IYYVCV** 1035

CaDoa10 IIILPVLLFAVVLGRPILRA--NLIIIDQL-------PHINIDELTNMDWRLTDLASIAI 920

* * .. ... ... . .. . : .* :: . .: . .. .

HsTEB4 GRRVIFQKVKEWSLMI--------------------------------MKTLIVAVLLAG 729

ScDoa10 **GSVCLSKIAKDILHFTE**GQNTLDEHAVDENEVEEVEHDIPERDINNAPVNNINNVEEGQG 1095

CaDoa10 G---LAVELQALIYYD----------------KNFAADAP----NNAGGNEIQQADPVRG 957

* : .:. . : . *

HsTEB4 VVP--LLLGLLFELVIV--------APLRVPLDQTPLFYPWQDWALGVL------HAKII 773

ScDoa10 IFM--AIFNSIFDS**MLVKYNLMVFIAIM---IAVIRTMVSWVVLTDGILACYNYLTI**RVF 1150

CaDoa10 MLLQGNLLNQAFNRLP---------APV---LYTLPSLMLWIMWILTVH------KLCVD 999

.. ::. *. . *.. : .. . * ....:. :

HsTEB4 AAITLM-GP-------------QWWLKTVIEQVYANGIRNIDLHY---------IVRKLA 810

ScDoa10 GNSSYTIGNSKWFKYD**ESLLFVVWIISSMVN--FGTGYKSLKLFFRN**RNTS**KLNFLKTMA** 1208

CaDoa10 QPIRYMTGN---LHVEFLLNFKTLLIHFLVS--FWTILPA--LVY---------VTRRIP 1043

. .. *. . : .: : .. . * : . :...

HsTEB4 APVISVLL-LSLC--VPYVIASGVVPLLG-VTAEMQNLVHRR------------IYPFLL 854

ScDoa10 **LELFKQGF-LHMVIYVLPIIILSLVFLR**DVSTKQIIDISHGSRSFTLSLNESFPTWTRMQ 1267

CaDoa10 VEGQTAWQTLKRCGIIPCLLNFSMVHIPAFIVLYSLKVLDKA-----NISVSFYIWPVLF 1098

. * . :. .: ..* : .. .. .. ... :

HsTEB4 MVV--VLMAILSFQ---------VRQFKRLYEHIKNDKYLVGQRLVNYERKSGKQGSSPP 903

ScDoa10 D**IYFGLLIALESFTFFFQATVLFIQWFKSTVQNVKDEVY**TKGRALENLPDES-------- 1319

CaDoa10 ICF--ALVKLITEG---------AKLYTNINNQVKQEKYVKGRAIENVEDDD-------- 1139

.*..: . .. :. . ..:*.:.* .*:.:.* ....

HsTEB4 PPQSSQE 910

ScDoa10 ------- 1320

CaDoa10 ------- 1140

**KEY**

**Red bold** = RING domain

**Green bold** = Metal (zinc)-coordinating Cys and His residues in RING domain

Yellow highlight = Trp commonly found in RING domains

Gray highlight = Transmembrane portion of protein

**Black bold** = approximate positions of 14 TM segments of Sc Doa10 per AlphaFold (Mehrtash and Hochstrasser 2022)

Underlined = TEB4-Doa10 (TD) Domain

Turqoise highlight = Sc C-terminal extension (activates E2 Ubc6, per Mehrtash and Hochstrasser 2022)

Produced using SnapGene.
