# Supplementary material for: Characterization of endoplasmic reticulum-associated degradation in the human fungal pathogen Candida albicans
Source: PeerJ. 2023 Aug 25;11:e15897. doi: 10.7717/peerj.15897 (PMC10461541; doi:10.7717/peerj.15897)
Supplement: Supplemental Information 4 [file peerj-11-15897-s004.docx]

**Table S1.** Samples and TMTpro barcodes.

| **Sample** | **TMTpro label** |
| --- | --- |
| Wild Type #1 | 126 |
| Wild Type #2 | 127N |
| Wild Type #3 | 127C |
| *doa10*/*doa10* #1 | 128N |
| *doa10*/*doa10* #2 | 128C |
| *doa10*/*doa10* #3 | 129N |
| *hrd1*/*hrd1* #1 | 129C |
| *hrd1*/*hrd1* #2 | 13N |
| *hrd1*/*hrd1* #3 | 132C |
| *ubc7*/*ubc7* #1 | 133N |
| *ubc7*/*ubc7* #2 | 133C |
| *ubc7*/*ubc7* #3 | 134N |
