# Supplementary material for: Characterization of endoplasmic reticulum-associated degradation in the human fungal pathogen Candida albicans
Source: PeerJ. 2023 Aug 25;11:e15897. doi: 10.7717/peerj.15897 (PMC10461541; doi:10.7717/peerj.15897)
Supplement: Supplemental Information 5 — Full alignments are available in Supplemental File 1 (Hrd1), Supplemental File 2 (Doa10), and Figure 2D (Ubc7). [file peerj-11-15897-s005.docx]

**Table 2.** Sequence identity and similarity of *C. albicans*, *S. cerevisiae*, and *H. sapiens* homologs of ERAD enzymes Hrd1, Doa10, and Ubc7.

|  | *S. cerevisiae* | *H. sapiens* |
| --- | --- | --- |
| *C. albicans* Hrd1 | 25.0 % identical to ScHrd1  43.0 % similar to ScHrd1 | 21.7 % identical to HsHRD1  36.4 % similar to HsHRD1 |
| *C. albicans* Doa10 | 27.2 % identical to ScDoa10  45.2 % similar to ScDoa10 | 24.8 % identical to HsMARCHF6  40.0 % similar to HsMARCHF6 |
| *C. albicans* Ubc7 | 71.2 % identical to ScUbc7  83.4 % similar to ScUbc7 | 63.0 % identical to HsUBE2G2  80.9 % similar to HsUBE2G2 |
| *S. cerevisiae* Hrd1 |  | 25.3 % identical to HsHRD1  43.3 % similar to HsHRD1 |
| *S. cerevisiae* Doa10 |  | 21.3 % identical to HsMARCHF6  36.2 % similar to HsMARCHF6 |
| *S. cerevisiae* Ubc7 |  | 62.8 % identical to HsUBE2G2  75.0 % identical to HsUBE2G2 |

Full alignments are available in Supplemental File 1 (Hrd1), Supplemental File 2 (Doa10), and Figure 2D (Ubc7).
