# Supplementary material for: Characterization of endoplasmic reticulum-associated degradation in the human fungal pathogen Candida albicans
Source: PeerJ. 2023 Aug 25;11:e15897. doi: 10.7717/peerj.15897 (PMC10461541; doi:10.7717/peerj.15897)
Supplement: Supplemental Information 6 [file peerj-11-15897-s006.docx]

**Table S3.** Proteins with significant increases in abundance in multiple *C. albicans* mutants.

| **Protein** | **Genotypes exhibiting significant increase in abundance** |
| --- | --- |
| Pyc2 | *hrd1*/*hrd1 doa10*/*doa10*  *ubc7*/*ubc7* |
| Arf3 | *hrd1*/*hrd1 ubc7*/*ubc7* |
| Dck1 | *hrd1*/*hrd1 ubc7*/*ubc7* |
| Dpm1 | *hrd1*/*hrd1 ubc7*/*ubc7* |
| Erg3 | *hrd1*/*hrd1 ubc7*/*ubc7* |
| Gdh3 | *hrd1*/*hrd1 ubc7*/*ubc7* |
| His1 | *hrd1*/*hrd1 ubc7*/*ubc7* |
| Kar2 | *hrd1*/*hrd1 ubc7*/*ubc7* |
| Mrp8 | *hrd1*/*hrd1 ubc7*/*ubc7* |
| Pdi1 | *hrd1*/*hrd1 ubc7*/*ubc7* |
| Pga63 | *hrd1*/*hrd1 ubc7*/*ubc7* |
| Rot1 | *hrd1*/*hrd1 ubc7*/*ubc7* |
| Sec24 | *hrd1*/*hrd1 ubc7*/*ubc7* |
| Sec61 | *hrd1*/*hrd1 ubc7*/*ubc7* |
| Ser1 | *hrd1*/*hrd1 ubc7*/*ubc7* |
| Taf145 | *hrd1*/*hrd1 ubc7*/*ubc7* |
| C6_00270W | *hrd1*/*hrd1 ubc7*/*ubc7* |
| C4_05390W | *hrd1*/*hrd1 ubc7*/*ubc7* |
| C3_02620C | *hrd1*/*hrd1 ubc7*/*ubc7* |
| C7_01350C | *hrd1*/*hrd1 ubc7*/*ubc7* |
| C2_03450W | *hrd1*/*hrd1 ubc7*/*ubc7* |
| Hmx1 | *doa10*/*doa10 ubc7*/*ubc7* |
| Lys9 | *doa10*/*doa10 ubc7*/*ubc7* |
| Pex5 | *doa10*/*doa10 ubc7*/*ubc7* |
| Rct1 | *hrd1*/*hrd1 doa10*/*doa10* |
