# Supplementary material for: Characterization of endoplasmic reticulum-associated degradation in the human fungal pathogen Candida albicans
Source: PeerJ. 2023 Aug 25;11:e15897. doi: 10.7717/peerj.15897 (PMC10461541; doi:10.7717/peerj.15897)
Supplement: Supplemental Information 7 [file peerj-11-15897-s007.docx]

**Table S4.** Proteins with significant decreases in abundance in multiple *C. albicans* mutants.

| **Protein** | **Genotypes exhibiting significant decrease in abundance** |
| --- | --- |
| CR_07220C | *hrd1*/*hrd1 doa10*/*doa10*  *ubc7*/*ubc7* |
| Eno1 | *hrd1*/*hrd1 doa10*/*doa10*  *ubc7*/*ubc7* |
| Med18 | *hrd1*/*hrd1 doa10*/*doa10*  *ubc7*/*ubc7* |
| Adp1 | *hrd1*/*hrd1 ubc7*/*ubc7* |
| Ino1 | *hrd1*/*hrd1 ubc7*/*ubc7* |
| Rpt2 | *hrd1*/*hrd1 ubc7*/*ubc7* |
| Sap190 | *hrd1*/*hrd1 ubc7*/*ubc7* |
| Yrb1 | *hrd1*/*hrd1 ubc7*/*ubc7* |
| C2_00060C | *hrd1*/*hrd1 ubc7*/*ubc7* |
| C1_12070C | *hrd1*/*hrd1 ubc7*/*ubc7* |
| C1_04790W | *hrd1*/*hrd1 ubc7*/*ubc7* |
