# Supplementary material for: Characterization of endoplasmic reticulum-associated degradation in the human fungal pathogen Candida albicans
Source: PeerJ. 2023 Aug 25;11:e15897. doi: 10.7717/peerj.15897 (PMC10461541; doi:10.7717/peerj.15897)
Supplement: Supplemental Information 8 [file peerj-11-15897-s008.docx]

**Table S5.** Proteins with significant, opposite-direction changes in abundance in multiple *C. albicans* mutants.

| **Protein** | **Genotypes exhibiting significant change in abundance** |
| --- | --- |
| Gdh3 | Down in *doa10/doa10* Up in *ubc7*/*ubc7* Up in *hrd1*/*hrd1* |
| orf19.4639 | Down in *doa10/doa10* Up in *hrd1*/*hrd1* |
| Ade2 | Down in *doa10/doa10* Up in *ubc7/ubc7* |
| Age1 | Down in *doa10/doa10* Up in *ubc7/ubc7* |
| Rpl18 | Down in *doa10/doa10* Up in *ubc7/ubc7* |
| Spe3 | Down in *doa10/doa10* Up in *ubc7/ubc7* |
| Erg8 | Up in *doa10/doa10* Down in *ubc7*/*ubc7* |
