# Supplementary material for: Characterization of endoplasmic reticulum-associated degradation in the human fungal pathogen Candida albicans
Source: PeerJ. 2023 Aug 25;11:e15897. doi: 10.7717/peerj.15897 (PMC10461541; doi:10.7717/peerj.15897)
Supplement: Supplemental Information 9 — Genes encoding proteins with altered abundance in hrd1/hrd1 C. albicans mutants were analyzed using the Gene Ontology Term Finder at the Candida Genome Database ( http://www.candidagenome.org/cgi-bin/GO/goTermFinder ). No significant Process, Function, or Component GO Terms were found for proteins present in decreased abundance in hrd1/hrd1 mutants. [file peerj-11-15897-s009.docx]

**Table S6.** Gene Ontology Term analysis for proteins present in increased abundance in *C. albicans* *hrd1*/*hrd1* mutants.

|  | **GO Term** | **Corrected p value** | **FDR** | **Genes** |
| --- | --- | --- | --- | --- |
| Process | cell wall biogenesis | 0.02568 | 22.00% | *ROT1 DPM1 CCR4 KAR2 PMI1 PMT1* |
|  | small molecule biosynthetic process | 0.03567 | 12.00% | *ERG3 PGA63 DPM1 SEC24 AAT1 SER1 SEC61 PYC2 GDH3 HIS1* |
|  | ERAD pathway | 0.06133 | 10.67% | *KAR2 SEC61 PMT1 PDI1* |
|  | protein O-linked glycosylation | 0.06847 | 8.00% | *ROT1 DPM1 PMT1* |
|  | fungal-type cell wall biogenesis | 0.0873 | 8.00% | *ROT1 DPM1 CCR4 KAR2 PMI1* |
| Function | vitamin binding | 0.01564 | 24.00% | *GLY1 AAT1 SER1 PYC2* |
|  | unfolded protein binding | 0.05838 | 25.00% | *ROT1 KAR2 C7_01350C PDI1* |
| Component | endoplasmic reticulum lumen | 0.00274 | 0.00% | *ERG3 KAR2 PDI1* |
|  | endoplasmic reticulum | 0.00634 | 0.00% | *ROT1 ERG3 DPM1 SEC24 KAR2 SEC61 C6_00270W PMT1 PDI1* |
|  | cytoplasm | 0.07606 | 3.33% | *ROT1 ERG3 RPS27A PGA63 TAF145 DPM1 CCR4 SEC24 SEC18 KAR2 DCK1 AAT1 C3_02620C SEC61 C4_03200C MSN5 PYC2 GDH3 C4_06690C HIS1 C6_00270W C7_01350C PMT1 PDI1 TLO16* |
|  | COPII vesicle coat | 0.07634 | 3.50% | *PGA63 SEC24* |
|  | endoplasmic reticulum protein-containing complex | 0.08196 | 2.80% | *KAR2 SEC61 C6_00270W PMT1* |

Genes encoding proteins with altered abundance in *hrd1*/*hrd1 C. albicans* mutants were analyzed using the Gene Ontology Term Finder at the Candida Genome Database (<http://www.candidagenome.org/cgi-bin/GO/goTermFinder>). No significant Process, Function, or Component GO Terms were found for proteins present in decreased abundance in *hrd1*/*hrd1* mutants.
