# Supplementary material for: Characterization of endoplasmic reticulum-associated degradation in the human fungal pathogen Candida albicans
Source: PeerJ. 2023 Aug 25;11:e15897. doi: 10.7717/peerj.15897 (PMC10461541; doi:10.7717/peerj.15897)
Supplement: Supplemental Information 10 — Genes encoding proteins with altered abundance in doa10/doa10 C. albicans mutants were analyzed using the Gene Ontology Term Finder at the Candida Genome Database ( http://www.candidagenome.org/cgi-bin/GO/goTermFinder ). No significant Process, Function, or Component GO Terms were found for proteins present in increased abundance in doa10/doa10 mutants. No significant Function or Component GO Terms were found for proteins present in decreased abundance in doa10/doa10 mutants. [file peerj-11-15897-s010.docx]

**Table S7.** Gene Ontology Term analysis for proteins present in decreased abundance in *C. albicans doa10*/*doa10* mutants.

|  | **GO Term** | **Corrected p value** | **FDR** | **Genes** |
| --- | --- | --- | --- | --- |
| Process | carboxylic acid metabolic process | 0.02783 | 8.00% | *ENO1 ALD5 IDH2 SPE3 HIS4 CAR2 GDH3* |
|  | oxoacid metabolic process | 0.03461 | 5.00% | *ENO1 ALD5 IDH2 SPE3 HIS4 CAR2 GDH3* |
|  | organic acid metabolic process | 0.03846 | 3.33% | *ENO1 ALD5 IDH2 SPE3 HIS4 CAR2 GDH3* |

Genes encoding proteins with altered abundance in *doa10*/*doa10 C. albicans* mutants were analyzed using the Gene Ontology Term Finder at the Candida Genome Database (<http://www.candidagenome.org/cgi-bin/GO/goTermFinder>). No significant Process, Function, or Component GO Terms were found for proteins present in increased abundance in *doa10*/*doa10* mutants. No significant Function or Component GO Terms were found for proteins present in decreased abundance in *doa10*/*doa10* mutants.
