# Supplementary material for: Characterization of endoplasmic reticulum-associated degradation in the human fungal pathogen Candida albicans
Source: PeerJ. 2023 Aug 25;11:e15897. doi: 10.7717/peerj.15897 (PMC10461541; doi:10.7717/peerj.15897)
Supplement: Supplemental Information 11 — Genes encoding proteins with altered abundance in ubc7/ubc7 C. albicans mutants were analyzed using the Gene Ontology Term Finder at the Candida Genome Database ( http://www.candidagenome.org/cgi-bin/GO/goTermFinder ). No significant Process, Function, or Component GO Terms were found for proteins present in decreased abundance in ubc7/ubc7 mutants. [file peerj-11-15897-s011.docx]

**Table S8.** Gene Ontology Term analysis for proteins present in increased abundance in *C. albicans ubc7*/*ubc7*mutants.

|  | **GO Term** | **Corrected p value** | **FDR** | **Genes** |
| --- | --- | --- | --- | --- |
| Process | small molecule metabolic process | 1.01E-05 | 0.00% | *MVD POR1 ERG3 PGA63 DPM1 SEC24 GAD1 FAD3 ERG10 SER1 SPE3 IPP1 C2_09970C ADE2 LYS9 SEC61 PEX5 PYC2 GDH3 ACH1 GRP2 HIS1 ERG5 C7_04210C ATP3 ERG25 LAP3 URA2* |
|  | small molecule biosynthetic process | 8.02E-05 | 0.00% | *POR1 ERG3 PGA63 DPM1 SEC24 FAD3 ERG10 SER1 SPE3 IPP1 LYS9 SEC61 PEX5 PYC2 GDH3 HIS1 ERG5 C7_04210C ERG25* |
|  | organic substance biosynthetic process | 0.00023 | 0.00% | *MVD ROT1 TIM21 POR1 ERG3 PGA63 TAF145 DPM1 SEC24 MIR1 RPL2 MRPS9 FAD3 KAR2 ERG10 NDH51 SER1 SPE3 C2_07680W IPP1 ADE2 C3_05280C LYS9 SEC61 PEX5 PYC2 GDH3 TIF5 SUI1 C5_04530W HIS1 C6_00270W YHM1 C6_01980C C6_02460C C7_01350C TOM40 C7_02120C ERG5 C7_04210C ATP3 ERG25 URA2 CR_10830C* |
|  | biosynthetic process | 0.00036 | 0.00% | *MVD ROT1 TIM21 POR1 ERG3 PGA63 TAF145 DPM1 SEC24 MIR1 RPL2 MRPS9 FAD3 KAR2 ERG10 NDH51 SER1 SPE3 C2_07680W IPP1 ADE2 C3_05280C LYS9 SEC61 PEX5 PYC2 GDH3 TIF5 SUI1 C5_04530W HIS1 C6_00270W YHM1 C6_01980C C6_02460C C7_01350C TOM40 C7_02120C ERG5 C7_04210C ATP3 ERG25 URA2 CR_10830C* |
|  | cellular biosynthetic process | 0.00052 | 0.00% | *MVD ROT1 TIM21 POR1 ERG3 PGA63 TAF145 DPM1 SEC24 MIR1 RPL2 MRPS9 FAD3 KAR2 ERG10 NDH51 SER1 SPE3 C2_07680W IPP1 ADE2 C3_05280C LYS9 SEC61 PEX5 GDH3 TIF5 SUI1 C5_04530W HIS1 C6_00270W YHM1 C6_01980C C6_02460C C7_01350C TOM40 C7_02120C ERG5 C7_04210C ATP3 ERG25 URA2 CR_10830C* |
|  | cellular process | 0.00057 | 0.00% | *MVD HMX1 ROT1 C1_01160C TIM21 AGE1 C1_03830C POR1 ERG3 C1_05490C C1_06540C PGA63 TAF145 DPM1 SEC24 MIR1 RPL2 GAD1 MRPS9 FAD3 DIM1 KAR2 C2_02800W C2_03450W DCK1 ERG10 NDH51 RPF2 SER1 SPE3 C2_07680W IPP1 C2_09970C HOS2 NCE103 ARF3 C3_02620C ADE2 SFP1 C3_05150W C3_05280C LYS9 SEC61 PEX5 PYC2 C4_05390W C4_05820W GDH3 ACH1 TIF5 TRR1 GRP2 SUI1 C5_04530W TIP20 HIS1 C6_00270W C6_00340C YHM1 C6_01980C C6_02460C SOD3 C7_01350C TOM40 C7_02120C TOM70 ERG5 PDI1 C7_04210C ATP3 ERG25 RFG1 LAP3 HSP60 URA2 ACO1 CR_10830C* |
|  | cellular amide metabolic process | 0.00076 | 0.00% | *MVD TIM21 MIR1 RPL2 MRPS9 KAR2 NDH51 SER1 SPE3 C2_07680W IPP1 C3_05280C PEX5 ACH1 TIF5 SUI1 C5_04530W YHM1 C6_01980C C6_02460C C7_01350C TOM40 C7_02120C ERG5 ATP3 ERG25 URA2 CR_10830C* |
|  | primary metabolic process | 0.00076 | 0.00% | *MVD ROT1 C1_01160C TIM21 C1_03830C POR1 ERG3 C1_06540C PGA63 TAF145 DPM1 SEC24 MIR1 RPL2 GAD1 MRPS9 FAD3 DIM1 KAR2 C2_03450W ERG10 NDH51 RPF2 SER1 SPE3 C2_07680W IPP1 C2_09970C HOS2 NCE103 ADE2 SFP1 C3_05150W C3_05280C LYS9 SEC61 PEX5 PYC2 C4_05390W C4_05820W GDH3 ACH1 TIF5 TRR1 SUI1 C5_04530W TIP20 HIS1 C6_00270W C6_00340C YHM1 C6_01980C C6_02460C C7_01350C TOM40 C7_02120C ERG5 PDI1 C7_04210C ATP3 ERG25 LAP3 HSP60 URA2 ACO1 CR_10830C* |
|  | organic substance metabolic process | 0.00103 | 0.00% | *MVD HMX1 ROT1 C1_01160C TIM21 C1_03830C POR1 ERG3 C1_06540C PGA63 TAF145 DPM1 SEC24 MIR1 RPL2 GAD1 MRPS9 FAD3 DIM1 KAR2 C2_03450W ERG10 NDH51 RPF2 SER1 SPE3 C2_07680W IPP1 C2_09970C HOS2 NCE103 ADE2 SFP1 C3_05150W C3_05280C LYS9 SEC61 PEX5 PYC2 C4_05390W C4_05820W GDH3 ACH1 TIF5 TRR1 GRP2 SUI1 C5_04530W TIP20 HIS1 C6_00270W C6_00340C YHM1 C6_01980C C6_02460C C7_01350C TOM40 C7_02120C ERG5 PDI1 C7_04210C ATP3 ERG25 LAP3 HSP60 URA2 CR_10830C* |
|  | intracellular protein transmembrane transport | 0.00114 | 0.00% | *TIM21 KAR2 C3_02620C SEC61 PEX5 C4_05820W TOM40 TOM70 HSP60 ACO1* |
|  | protein transmembrane transport | 0.00114 | 0.00% | *TIM21 KAR2 C3_02620C SEC61 PEX5 C4_05820W TOM40 TOM70 HSP60 ACO1* |
|  | organonitrogen compound biosynthetic process | 0.00153 | 0.00% | *ROT1 TIM21 DPM1 MIR1 RPL2 MRPS9 KAR2 NDH51 SER1 SPE3 C2_07680W IPP1 ADE2 C3_05280C LYS9 PEX5 GDH3 TIF5 SUI1 C5_04530W HIS1 C6_00270W YHM1 C6_01980C C6_02460C C7_01350C TOM40 C7_02120C ERG5 C7_04210C ATP3 ERG25 URA2 CR_10830C* |
|  | carboxylic acid metabolic process | 0.00177 | 0.00% | *ERG3 GAD1 FAD3 SER1 SPE3 LYS9 PEX5 PYC2 GDH3 ACH1 GRP2 HIS1 C7_04210C ERG25 LAP3 URA2* |
|  | amide biosynthetic process | 0.00246 | 0.00% | *TIM21 MIR1 RPL2 MRPS9 KAR2 NDH51 SER1 SPE3 C2_07680W IPP1 C3_05280C PEX5 TIF5 SUI1 C5_04530W YHM1 C6_01980C C6_02460C C7_01350C TOM40 C7_02120C ERG5 ATP3 ERG25 URA2 CR_10830C* |
|  | oxoacid metabolic process | 0.00278 | 0.00% | *ERG3 GAD1 FAD3 SER1 SPE3 LYS9 PEX5 PYC2 GDH3 ACH1 GRP2 HIS1 C7_04210C ERG25 LAP3 URA2* |
|  | sterol biosynthetic process | 0.00287 | 0.00% | *MVD POR1 ERG3 PGA63 DPM1 SEC24 ERG10 IPP1 SEC61 ERG5 ERG25* |
|  | steroid biosynthetic process | 0.00287 | 0.00% | *MVD POR1 ERG3 PGA63 DPM1 SEC24 ERG10 IPP1 SEC61 ERG5 ERG25* |
|  | organic acid metabolic process | 0.00345 | 0.00% | *ERG3 GAD1 FAD3 SER1 SPE3 LYS9 PEX5 PYC2 GDH3 ACH1 GRP2 HIS1 C7_04210C ERG25 LAP3 URA2* |
|  | translation | 0.00372 | 0.00% | *TIM21 MIR1 RPL2 MRPS9 KAR2 NDH51 SER1 SPE3 C2_07680W IPP1 C3_05280C TIF5 SUI1 C5_04530W YHM1 C6_01980C C6_02460C C7_01350C TOM40 C7_02120C ERG5 ATP3 ERG25 URA2 CR_10830C* |
|  | peptide biosynthetic process | 0.00398 | 0.00% | *TIM21 MIR1 RPL2 MRPS9 KAR2 NDH51 SER1 SPE3 C2_07680W IPP1 C3_05280C TIF5 SUI1 C5_04530W YHM1 C6_01980C C6_02460C C7_01350C TOM40 C7_02120C ERG5 ATP3 ERG25 URA2 CR_10830C* |
|  | protein transmembrane import into intracellular organelle | 0.00411 | 0.00% | *TIM21 C3_02620C SEC61 PEX5 C4_05820W TOM40 TOM70 HSP60 ACO1* |
|  | cellular metabolic process | 0.00456 | 0.00% | *MVD HMX1 ROT1 C1_01160C TIM21 AGE1 C1_03830C POR1 ERG3 C1_06540C PGA63 TAF145 DPM1 SEC24 MIR1 RPL2 GAD1 MRPS9 FAD3 DIM1 KAR2 C2_03450W DCK1 ERG10 NDH51 RPF2 SER1 SPE3 C2_07680W IPP1 C2_09970C NCE103 ADE2 SFP1 C3_05150W C3_05280C LYS9 SEC61 PEX5 PYC2 C4_05390W C4_05820W GDH3 ACH1 TIF5 TRR1 GRP2 SUI1 C5_04530W TIP20 HIS1 C6_00270W YHM1 C6_01980C C6_02460C SOD3 C7_01350C TOM40 C7_02120C ERG5 PDI1 C7_04210C ATP3 ERG25 LAP3 URA2 ACO1 CR_10830C* |
|  | sterol metabolic process | 0.00506 | 0.00% | *MVD POR1 ERG3 PGA63 DPM1 SEC24 ERG10 IPP1 SEC61 ERG5 ERG25* |
|  | metabolic process | 0.00521 | 0.00% | *MVD HMX1 ROT1 C1_01160C TIM21 AGE1 C1_03830C POR1 ERG3 C1_06540C PGA63 TAF145 DPM1 SEC24 MIR1 RPL2 GAD1 MRPS9 FAD3 DIM1 KAR2 C2_03450W DCK1 ERG10 NDH51 RPF2 SER1 SPE3 C2_07680W IPP1 C2_09970C HOS2 NCE103 ADE2 SFP1 C3_05150W C3_05280C LYS9 SEC61 PEX5 PYC2 C4_05390W C4_05820W GDH3 ACH1 TIF5 TRR1 GRP2 SUI1 C5_04530W TIP20 HIS1 C6_00270W C6_00340C YHM1 C6_01980C C6_02460C SOD3 C7_01350C TOM40 C7_02120C ERG5 PDI1 C7_04210C ATP3 ERG25 LAP3 HSP60 URA2 ACO1 CR_10830C* |
|  | steroid metabolic process | 0.00564 | 0.00% | *MVD POR1 ERG3 PGA63 DPM1 SEC24 ERG10 IPP1 SEC61 ERG5 ERG25* |
|  | peptide metabolic process | 0.00753 | 0.00% | *TIM21 MIR1 RPL2 MRPS9 KAR2 NDH51 SER1 SPE3 C2_07680W IPP1 C3_05280C TIF5 SUI1 C5_04530W YHM1 C6_01980C C6_02460C C7_01350C TOM40 C7_02120C ERG5 ATP3 ERG25 URA2 CR_10830C* |
|  | carboxylic acid biosynthetic process | 0.01049 | 0.07% | *ERG3 FAD3 SER1 SPE3 LYS9 PEX5 GDH3 HIS1 C7_04210C ERG25* |
|  | cellular macromolecule biosynthetic process | 0.01203 | 0.07% | *ROT1 TIM21 DPM1 MIR1 RPL2 MRPS9 KAR2 NDH51 SER1 SPE3 C2_07680W IPP1 C3_05280C TIF5 SUI1 C5_04530W C6_00270W YHM1 C6_01980C C6_02460C C7_01350C TOM40 C7_02120C ERG5 ATP3 ERG25 URA2 CR_10830C* |
|  | phytosteroid biosynthetic process | 0.01539 | 0.07% | *POR1 ERG3 PGA63 DPM1 SEC24 ERG10 IPP1 SEC61 ERG5 ERG25* |
|  | secondary alcohol biosynthetic process | 0.01539 | 0.07% | *POR1 ERG3 PGA63 DPM1 SEC24 ERG10 IPP1 SEC61 ERG5 ERG25* |
|  | cellular alcohol biosynthetic process | 0.01539 | 0.06% | *POR1 ERG3 PGA63 DPM1 SEC24 ERG10 IPP1 SEC61 ERG5 ERG25* |
|  | ergosterol biosynthetic process | 0.01539 | 0.06% | *POR1 ERG3 PGA63 DPM1 SEC24 ERG10 IPP1 SEC61 ERG5 ERG25* |
|  | cellular lipid biosynthetic process | 0.01539 | 0.06% | *POR1 ERG3 PGA63 DPM1 SEC24 ERG10 IPP1 SEC61 ERG5 ERG25* |
|  | organic hydroxy compound biosynthetic process | 0.01542 | 0.06% | *MVD POR1 ERG3 PGA63 DPM1 SEC24 ERG10 IPP1 SEC61 ERG5 ERG25* |
|  | organic acid biosynthetic process | 0.01711 | 0.06% | *ERG3 FAD3 SER1 SPE3 LYS9 PEX5 GDH3 HIS1 C7_04210C ERG25* |
|  | phytosteroid metabolic process | 0.01802 | 0.11% | *POR1 ERG3 PGA63 DPM1 SEC24 ERG10 IPP1 SEC61 ERG5 ERG25* |
|  | cellular alcohol metabolic process | 0.01802 | 0.11% | *POR1 ERG3 PGA63 DPM1 SEC24 ERG10 IPP1 SEC61 ERG5 ERG25* |
|  | ergosterol metabolic process | 0.01802 | 0.11% | *POR1 ERG3 PGA63 DPM1 SEC24 ERG10 IPP1 SEC61 ERG5 ERG25* |
|  | secondary alcohol metabolic process | 0.02213 | 0.10% | *POR1 ERG3 PGA63 DPM1 SEC24 ERG10 IPP1 SEC61 ERG5 ERG25* |
|  | alcohol biosynthetic process | 0.052 | 0.25% | *POR1 ERG3 PGA63 DPM1 SEC24 ERG10 IPP1 SEC61 ERG5 ERG25* |
|  | protein targeting to mitochondrion | 0.05488 | 0.24% | *TIM21 C3_02620C C4_05820W C6_00340C TOM40 TOM70 HSP60 ACO1* |
|  | protein import into mitochondrial matrix | 0.06458 | 0.29% | *TIM21 C3_02620C C4_05820W TOM40 TOM70 HSP60 ACO1* |
|  | organonitrogen compound metabolic process | 0.07465 | 0.47% | *MVD HMX1 ROT1 TIM21 DPM1 MIR1 RPL2 GAD1 MRPS9 KAR2 NDH51 SER1 SPE3 C2_07680W IPP1 C2_09970C HOS2 ADE2 C3_05280C LYS9 SEC61 PEX5 C4_05390W C4_05820W GDH3 ACH1 TIF5 TRR1 SUI1 C5_04530W TIP20 HIS1 C6_00270W C6_00340C YHM1 C6_01980C C6_02460C C7_01350C TOM40 C7_02120C ERG5 PDI1 C7_04210C ATP3 ERG25 LAP3 HSP60 URA2 CR_10830C* |
|  | protein localization to mitochondrion | 0.07741 | 0.45% | *TIM21 C3_02620C C4_05820W C6_00340C TOM40 TOM70 HSP60 ACO1* |
|  | establishment of protein localization to mitochondrion | 0.07741 | 0.44% | *TIM21 C3_02620C C4_05820W C6_00340C TOM40 TOM70 HSP60 ACO1* |
| Function | oxidoreductase activity, acting on paired donors, with oxidation of a pair of donors resulting in the reduction of molecular oxygen to two molecules of water | 0.00813 | 2.00% | *ERG3 FAD3 ERG5* |
|  | amide binding | 0.01649 | 1.00% | *SEC24 SEC61 PEX5 PYC2 TOM70* |
|  | sterol desaturase activity | 0.0195 | 1.33% | *ERG3 ERG5* |
|  | signal sequence binding | 0.02169 | 1.00% | *SEC24 SEC61 PEX5 TOM70* |
|  | oxidoreductase activity, acting on paired donors, with incorporation or reduction of molecular oxygen | 0.03237 | 1.20% | *HMX1 ERG3 FAD3 ERG5 ERG25* |
|  | peptide binding | 0.05491 | 2.33% | *SEC24 SEC61 PEX5 TOM70* |
| Component | cytoplasm | 2.60E-10 | 0.00% | *HMX1 ROT1 C1_01160C TIM21 AGE1 C1_03830C POR1 ERG3 C1_05490C C1_06540C PGA63 TAF145 DPM1 SEC24 MIR1 RPL2 MRPS9 KAR2 C2_02800W DCK1 ERG10 NDH51 C2_07680W IPP1 HOS2 NCE103 C3_02620C SFP1 RPL18 C3_05150W SEC61 PEX5 PYC2 C4_05820W GDH3 ACH1 TIF5 TRR1 SUI1 C5_04530W TIP20 HIS1 C6_00270W C6_00340C C6_01980C C6_02460C SOD3 C7_01350C TOM40 C7_02120C TOM70 ERG5 PDI1 ATP3 ERG25 LAP3 HSP60 URA2 ACO1 CR_10830C* |
|  | mitochondrion | 8.98E-06 | 0.00% | *TIM21 POR1 C1_05490C MIR1 MRPS9 DCK1 NDH51 C2_07680W NCE103 C3_02620C C4_05820W ACH1 TRR1 C5_04530W C6_00340C C6_01980C C6_02460C SOD3 C7_01350C TOM40 C7_02120C TOM70 ATP3 HSP60 ACO1 CR_10830C* |
|  | mitochondrial protein-containing complex | 0.00015 | 0.00% | *TIM21 C1_05490C MRPS9 NDH51 C2_07680W C5_04530W C6_01980C C6_02460C TOM40 C7_02120C TOM70 ATP3 CR_10830C* |
|  | intracellular anatomical structure | 0.00019 | 0.00% | *HMX1 ROT1 C1_01160C TIM21 AGE1 C1_03830C POR1 ERG3 C1_05490C C1_06540C PGA63 TAF145 DPM1 SEC24 MIR1 RPL2 MRPS9 KAR2 C2_02800W DCK1 ERG10 NDH51 RPF2 C2_07680W IPP1 C2_09970C HOS2 NCE103 ARF3 C3_02620C SFP1 RPL18 C3_05150W SEC61 PEX5 PYC2 C4_05820W GDH3 ACH1 TIF5 TRR1 SUI1 C5_04530W TIP20 HIS1 C6_00270W C6_00340C C6_01980C C6_02460C SOD3 C7_01350C TOM40 C7_02120C TOM70 ERG5 PDI1 ATP3 ERG25 LAP3 HSP60 URA2 ACO1 CR_10830C* |
|  | organelle membrane | 0.00154 | 0.00% | *HMX1 ROT1 TIM21 POR1 C1_05490C PGA63 SEC24 MIR1 KAR2 C2_02800W NDH51 C3_02620C SEC61 PEX5 C4_05820W C6_00270W C6_02460C TOM40 TOM70 ATP3 ERG25 HSP60* |
|  | mitochondrial envelope | 0.0016 | 0.00% | *TIM21 POR1 C1_05490C MIR1 NDH51 NCE103 C3_02620C C4_05820W TRR1 C6_02460C TOM40 TOM70 ATP3 HSP60* |
|  | membrane-bounded organelle | 0.00221 | 0.00% | *HMX1 ROT1 C1_01160C TIM21 AGE1 C1_03830C POR1 ERG3 C1_05490C C1_06540C PGA63 TAF145 DPM1 SEC24 MIR1 MRPS9 KAR2 C2_02800W DCK1 NDH51 RPF2 C2_07680W C2_09970C HOS2 NCE103 ARF3 C3_02620C SFP1 C3_05150W SEC61 PEX5 C4_05820W GDH3 ACH1 TRR1 C5_04530W TIP20 C6_00270W C6_00340C C6_01980C C6_02460C SOD3 C7_01350C TOM40 C7_02120C TOM70 ERG5 PDI1 ATP3 ERG25 HSP60 ACO1 CR_10830C* |
|  | organelle envelope | 0.00306 | 0.00% | *HMX1 TIM21 POR1 C1_05490C MIR1 KAR2 NDH51 NCE103 C3_02620C C4_05820W TRR1 C6_02460C TOM40 TOM70 ATP3 HSP60* |
|  | intracellular membrane-bounded organelle | 0.00316 | 0.00% | *HMX1 ROT1 C1_01160C TIM21 AGE1 C1_03830C POR1 ERG3 C1_05490C C1_06540C PGA63 TAF145 DPM1 SEC24 MIR1 MRPS9 KAR2 C2_02800W DCK1 NDH51 RPF2 C2_07680W C2_09970C HOS2 NCE103 C3_02620C SFP1 C3_05150W SEC61 PEX5 C4_05820W GDH3 ACH1 TRR1 C5_04530W TIP20 C6_00270W C6_00340C C6_01980C C6_02460C SOD3 C7_01350C TOM40 C7_02120C TOM70 ERG5 PDI1 ATP3 ERG25 HSP60 ACO1 CR_10830C* |
|  | envelope | 0.00325 | 0.00% | *HMX1 TIM21 POR1 C1_05490C MIR1 KAR2 NDH51 NCE103 C3_02620C C4_05820W TRR1 C6_02460C TOM40 TOM70 ATP3 HSP60* |
|  | integral component of mitochondrial membrane | 0.00402 | 0.00% | *POR1 C1_05490C MIR1 C3_02620C C6_02460C TOM70* |
|  | intrinsic component of mitochondrial membrane | 0.00505 | 0.00% | *POR1 C1_05490C MIR1 C3_02620C C6_02460C TOM70* |
|  | mitochondrial membrane | 0.00528 | 0.00% | *TIM21 POR1 C1_05490C MIR1 NDH51 C3_02620C C4_05820W C6_02460C TOM40 TOM70 ATP3 HSP60* |
|  | mitochondrial matrix | 0.00686 | 0.00% | *MRPS9 C2_07680W C4_05820W C5_04530W C6_00340C C6_01980C C6_02460C C7_02120C HSP60 ACO1 CR_10830C* |
|  | integral component of organelle membrane | 0.0088 | 0.00% | *POR1 C1_05490C MIR1 C2_02800W C3_02620C SEC61 C6_00270W C6_02460C TOM70* |
|  | endoplasmic reticulum | 0.00905 | 0.00% | *HMX1 ROT1 ERG3 DPM1 SEC24 KAR2 C2_02800W SEC61 TIP20 C6_00270W ERG5 PDI1 ERG25* |
|  | organellar ribosome | 0.00959 | 0.00% | *MRPS9 C2_07680W C5_04530W C6_01980C C6_02460C C7_02120C CR_10830C* |
|  | mitochondrial ribosome | 0.00959 | 0.00% | *MRPS9 C2_07680W C5_04530W C6_01980C C6_02460C C7_02120C CR_10830C* |
|  | intrinsic component of organelle membrane | 0.01337 | 0.00% | *POR1 C1_05490C MIR1 C2_02800W C3_02620C SEC61 C6_00270W C6_02460C TOM70* |
|  | organelle | 0.01379 | 0.00% | *HMX1 ROT1 C1_01160C TIM21 AGE1 C1_03830C POR1 ERG3 C1_05490C C1_06540C PGA63 TAF145 DPM1 SEC24 MIR1 RPL2 MRPS9 KAR2 C2_02800W DCK1 NDH51 RPF2 C2_07680W C2_09970C HOS2 NCE103 ARF3 C3_02620C SFP1 RPL18 C3_05150W SEC61 PEX5 C4_05820W GDH3 ACH1 TRR1 C5_04530W TIP20 C6_00270W C6_00340C C6_01980C C6_02460C SOD3 C7_01350C TOM40 C7_02120C TOM70 ERG5 PDI1 ATP3 ERG25 HSP60 ACO1 CR_10830C* |
|  | intracellular organelle | 0.01862 | 0.10% | *HMX1 ROT1 C1_01160C TIM21 AGE1 C1_03830C POR1 ERG3 C1_05490C C1_06540C PGA63 TAF145 DPM1 SEC24 MIR1 RPL2 MRPS9 KAR2 C2_02800W DCK1 NDH51 RPF2 C2_07680W C2_09970C HOS2 NCE103 C3_02620C SFP1 RPL18 C3_05150W SEC61 PEX5 C4_05820W GDH3 ACH1 TRR1 C5_04530W TIP20 C6_00270W C6_00340C C6_01980C C6_02460C SOD3 C7_01350C TOM40 C7_02120C TOM70 ERG5 PDI1 ATP3 ERG25 HSP60 ACO1 CR_10830C* |
|  | extracellular region | 0.01979 | 0.09% | *POR1 MIR1 KAR2 SPE3 IPP1 ARF3 SEC61 GDH3 GRP2 PDI1* |
|  | plasma membrane | 0.02334 | 0.09% | *HMX1 TIM21 AGE1 POR1 ERG3 DPM1 MIR1 DCK1 NDH51 SEC61 YHM1 TOM40 TOM70 ERG5 ATP3 ERG25* |
|  | endoplasmic reticulum lumen | 0.03455 | 0.08% | *ERG3 KAR2 PDI1* |
|  | nuclear exosome (RNase complex) | 0.04565 | 0.08% | *C1_01160C C1_03830C C1_06540C* |
|  | cytoplasmic exosome (RNase complex) | 0.04565 | 0.08% | *C1_01160C C1_03830C C1_06540C* |
|  | membrane | 0.05042 | 0.07% | *HMX1 ROT1 TIM21 AGE1 POR1 ERG3 C1_05490C PGA63 DPM1 SEC24 MIR1 KAR2 C2_02800W DCK1 NDH51 C3_02620C C3_05280C SEC61 PEX5 C4_05820W C6_00270W YHM1 C6_02460C TOM40 TOM70 ERG5 ATP3 ERG25 HSP60 URA2* |
|  | exosome (RNase complex) | 0.07417 | 0.36% | *C1_01160C C1_03830C C1_06540C* |
|  | membrane-enclosed lumen | 0.07845 | 0.34% | *C1_01160C C1_03830C ERG3 C1_06540C TAF145 MRPS9 KAR2 RPF2 C2_07680W HOS2 NCE103 C4_05820W TRR1 C5_04530W C6_00340C C6_01980C C6_02460C C7_02120C PDI1 HSP60 ACO1 CR_10830C* |
|  | organelle lumen | 0.07845 | 0.33% | *C1_01160C C1_03830C ERG3 C1_06540C TAF145 MRPS9 KAR2 RPF2 C2_07680W HOS2 NCE103 C4_05820W TRR1 C5_04530W C6_00340C C6_01980C C6_02460C C7_02120C PDI1 HSP60 ACO1 CR_10830C* |
|  | intracellular organelle lumen | 0.07845 | 0.32% | *C1_01160C C1_03830C ERG3 C1_06540C TAF145 MRPS9 KAR2 RPF2 C2_07680W HOS2 NCE103 C4_05820W TRR1 C5_04530W C6_00340C C6_01980C C6_02460C C7_02120C PDI1 HSP60 ACO1 CR_10830C* |
|  | ribosome | 0.07939 | 0.31% | *RPL2 MRPS9 C2_07680W RPL18 C5_04530W C6_01980C C6_02460C C7_02120C CR_10830C* |
|  | cell periphery | 0.08965 | 0.42% | *HMX1 TIM21 AGE1 POR1 ERG3 DPM1 MIR1 KAR2 DCK1 NDH51 IPP1 SEC61 TRR1 GRP2 YHM1 TOM40 TOM70 ERG5 ATP3 ERG25 ACO1* |
|  | exoribonuclease complex | 0.09186 | 0.47% | *C1_01160C C1_03830C C1_06540C* |

Genes encoding proteins with altered abundance in *ubc7*/*ubc7 C. albicans* mutants were analyzed using the Gene Ontology Term Finder at the Candida Genome Database (<http://www.candidagenome.org/cgi-bin/GO/goTermFinder>). No significant Process, Function, or Component GO Terms were found for proteins present in decreased abundance in *ubc7*/*ubc7* mutants.
